# Supplementary material for: Aerobic Degradation Characteristics and Mechanism of Decabromodiphenyl Ether (BDE-209) Using Complex Bacteria Communities
Source: Int J Environ Res Public Health. 2022 Dec 18;19(24):17012. doi: 10.3390/ijerph192417012 (PMC9778866; doi:10.3390/ijerph192417012)
Supplement: Supplementary file 1 [file ijerph-19-17012-s001.zip › ijerph-1983513-supplementary.pdf]

Supplementary materials for

Aerobic degradation characteristics and mechanism of decabromodiphenyl ether (BDE-209) using complex bacteria communities

Submitted to ***International Journal of Environmental Research and Public Health***

Dingfan Hu[†], Juan Wu[†,*], Luosheng Fan[†], Rong Jia[‡]

[†] School of Resources and Environmental Engineering, Anhui University, Hefei, Anhui 230601, P. R. China

[‡] School of Life Sciences, Anhui University, Hefei, Anhui 230601, P. R. China

*Corresponding author.

Address: No. 111 Kowloon Road, Shushan District, Heifei, 230601, Anhui Province, China.

Phone: +86-551-63861441. Fax: +86-551-63861724.

E-mail: wujuan@ustc.edu (J. Wu).

In total, there are two texts, one figure, and the document length is five pages.

Text S1 Analytical methods

1. Determination of BDE-209 concentration

The residual concentration of BDE-209 was analyzed by HPLC equipped with Agilent Eclipse Plus C18 column (250 × 4.6 mm, 5 μm). The column temperature and detection wavelength were set as 30°C and 240 nm, respectively. The mixture of acetonitrile and ultrapure water (98:2, V/V) was used as the mobile phase with 1.0 mL/min of flow rate, and the injection volume was 20 μL.

2. Analysis of degradation products

The intermediates during BDE-209 degradation were monitored and identified by LC-MS. The mobile phase consisted of 0.3% formic acid in water (A) and acetonitrile (B) with 0.2 mL/min of flow rate. The separation of intermediates was performed on a Thermo SCIENTIFIC Hypersil GOLD column (150 × 2.1 mm, 3 μm). The gradient elution procedure was as follows: 10% B (0-4 min), 10%-60% B (4-4.5 min, held for 3min), 60%-80% B (7.5-8 min, held for 3min), 80%-90% B (11-11.5 min, held for 3.5 min), 90%-100% B (15-15.5 min, held for 9.5 min), and 100%-10% B (25-25.5 min, held for 9.5 min). The MS spectrometer was operated with electrospray ionization (ESI) source in negative ion mode. Full scanning (m/z 50-1000 amu) was acquired under the following conditions: ionspray Voltage, -4500 V; temperature, 550°C; gas 1, 55 psi; gas 2, 55 psi; curtain gas, 35 psi; declustering potential, -80 V; collision Energy, -10 V.

Text S2

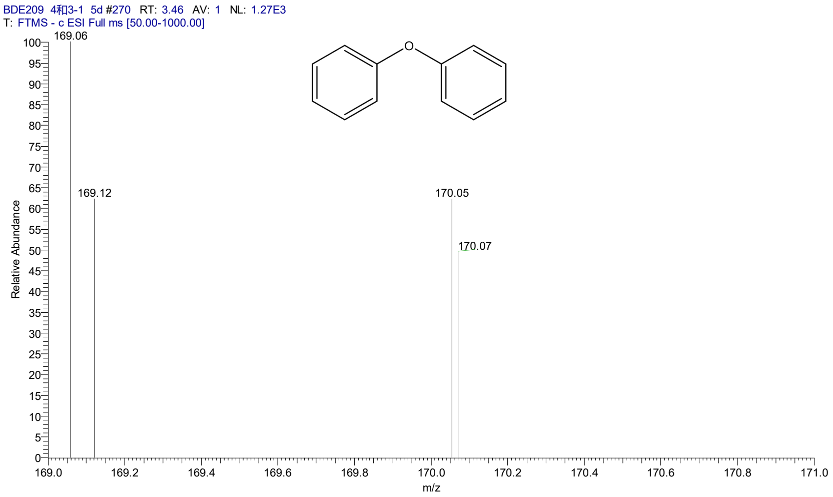

BDE209 4和3-1 5d #270 RT: 3.46 AV: 1 NL: 1.27E3
T: FTMS - c ESI Full ms [50.00-1000.00]
169.06
169.12
170.05
170.07
O
Relative Abundance
m/z

(a)

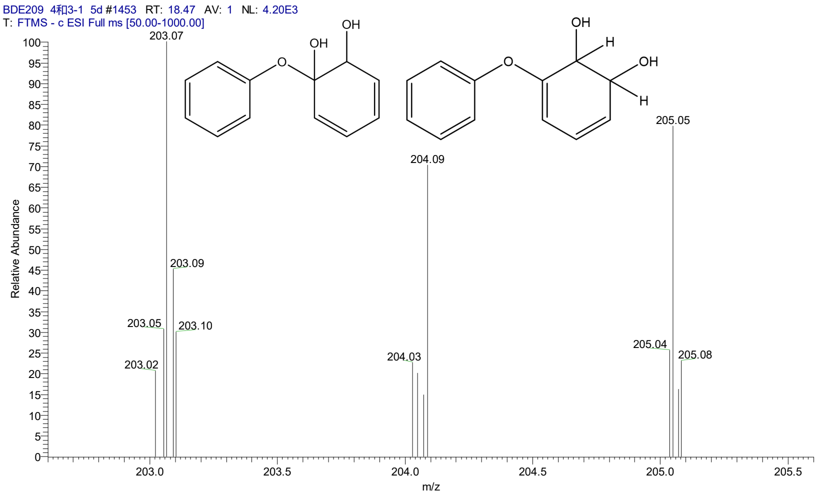

BDE209 4和3-1 5d #1453 RT: 18.47 AV: 1 NL: 4.20E3
T: FTMS - c ESI Full ms [50.00-1000.00]
203.07
203.09
203.05
203.10
203.02
204.09
204.03
205.05
205.04
205.08
O
OH
OH
H
H
Relative Abundance
m/z

(b、f)

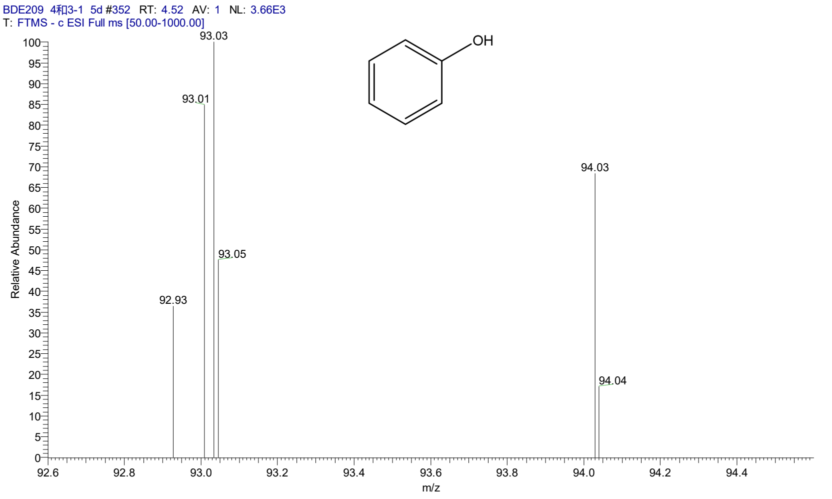

BDE209 4和3-1 5d #352 RT: 4.52 AV: 1 NL: 3.66E3
T: FTMS - c ESI Full ms [50.00-1000.00]
93.03
93.01
93.05
92.93
94.03
94.04
OH
Relative Abundance
m/z

(c)

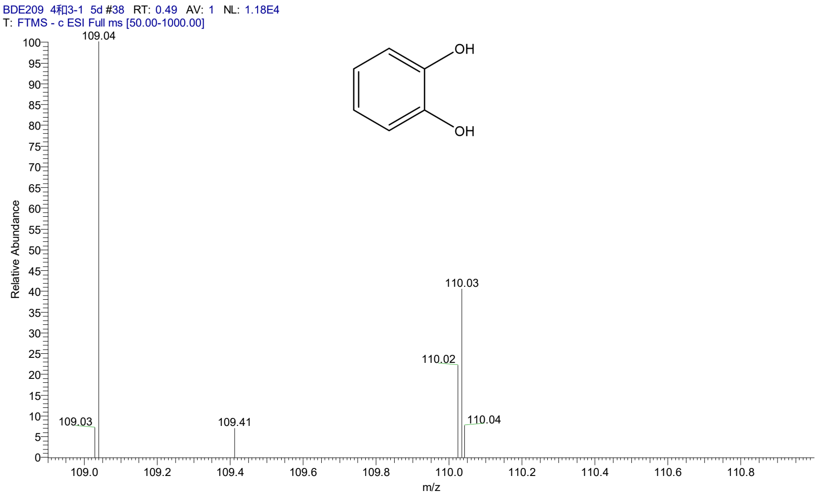

BDE209 4和3-1 5d #38 RT: 0.49 AV: 1 NL: 1.18E4
T: FTMS - c ESI Full ms [50.00-1000.00]
Relative Abundance
m/z
109.04
110.03
110.02
109.03
109.41
110.04
OH
OH

(d)

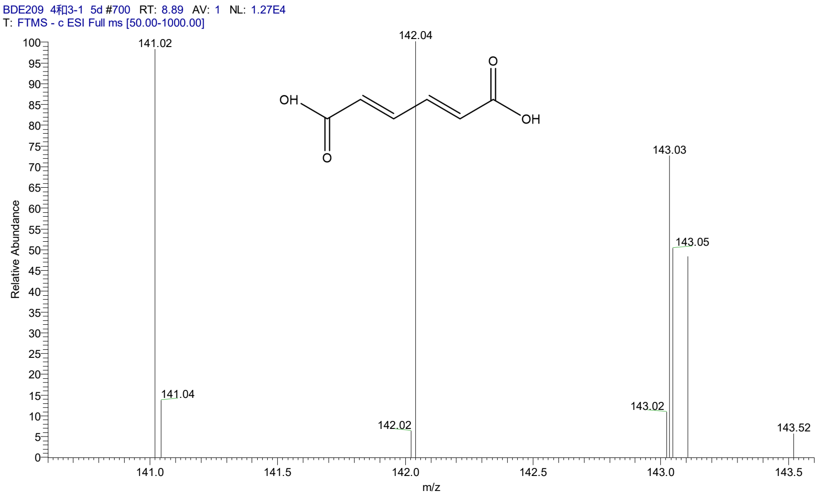

BDE209 4和3-1 5d #700 RT: 8.89 AV: 1 NL: 1.27E4
T: FTMS - c ESI Full ms [50.00-1000.00]
Relative Abundance
m/z
141.02
142.04
143.03
143.05
141.04
142.02
143.02
143.52
OH
O
OH
O

(e)

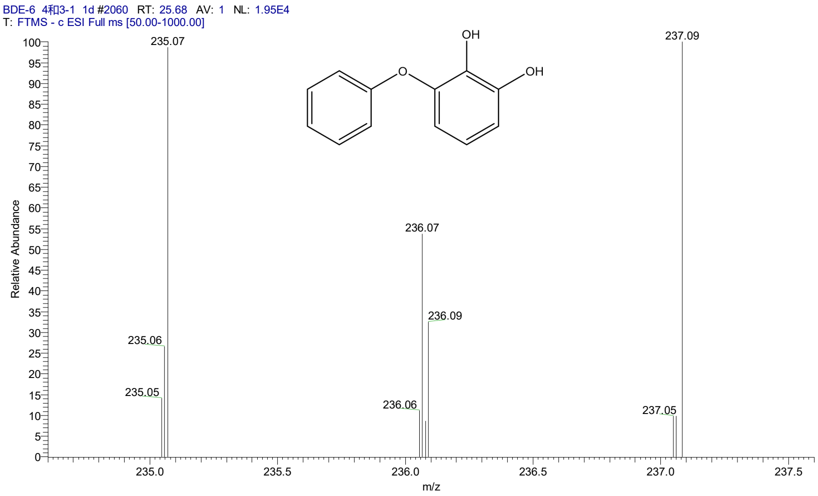

BDE-6 4和3-1 1d #2060 RT: 25.68 AV: 1 NL: 1.95E4
T: FTMS - c ESI Full ms [50.00-1000.00]
Relative Abundance
m/z
235.07
237.09
236.07
236.09
235.06
235.05
236.06
237.05
OH
OH
O

(g)

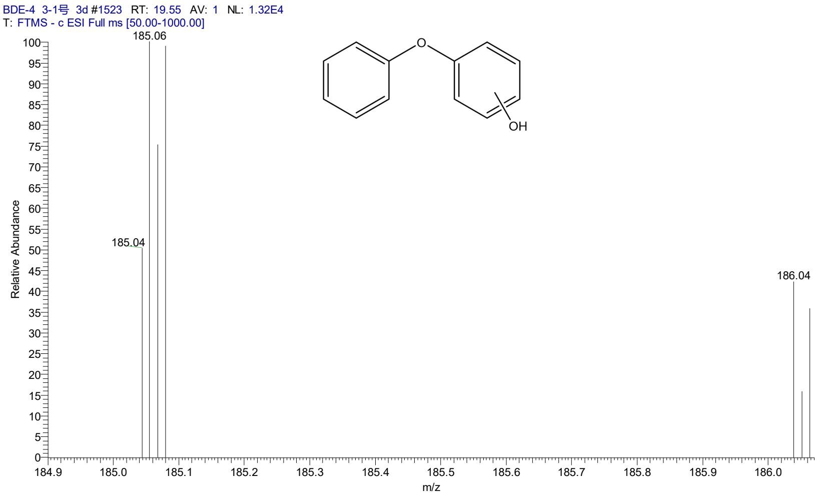

(h)

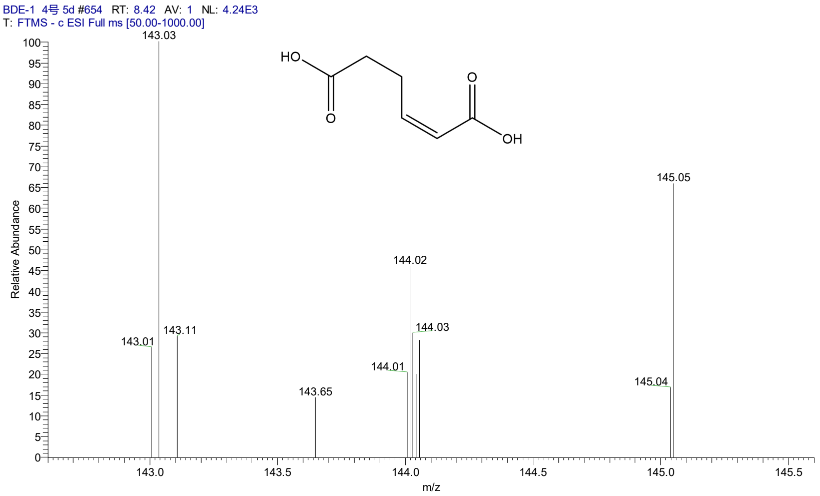

(i)

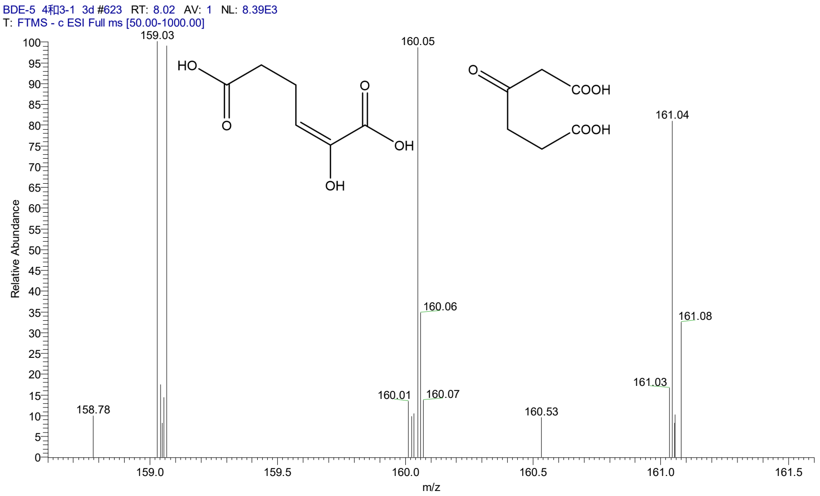

(j、k)

Figure S1. The mass spectra of intermediate products during the biodegradation of BDE-209 by compound bacteria M(1+2).

(The structures presented are representative examples of the products because the mass spectrum does not give information on the exact position of the hydroxyl group.)
